# Neural-Symbolic Ensemble Learning for early-stage prediction of critical state of Covid-19 patients

**DOI:** 10.1007/s11517-022-02674-1

**Published:** 2022-10-06

**Authors:** Arnaud Nguembang Fadja, Michele Fraccaroli, Alice Bizzarri, Giulia Mazzuchelli, Evelina Lamma

**Affiliations:** 1 Department of Mathematics and Computer Science, University of Ferrara, Via Nicolò Machiavelli 30, Ferrara, 44121 Italy; 2 DE - Department of Engineering, University of Ferrara, Via Saragat 1, Ferrara, 44122 Italy

**Keywords:** Covid-19, Decision Trees, Deep Learning, Hierarchical Probabilistic Logic Program, Severity

## Abstract

Recently, Artificial Intelligence (AI) and Machine Learning (ML) have been successfully applied to many domains of interest including medical diagnosis. Due to the availability of a large quantity of data, it is possible to build reliable AI systems that assist humans in making decisions. The recent Covid-19 pandemic quickly spread over the world causing serious health problems and severe economic and social damage. Computer scientists are actively working together with doctors on different ML models to diagnose Covid-19 patients using Computed Tomography (CT) scans and clinical data. In this work, we propose a neural-symbolic system that predicts if a Covid-19 patient arriving at the hospital will end in a critical condition. The proposed system relies on Deep 3D Convolutional Neural Networks (3D-CNNs) for analyzing lung CT scans of Covid-19 patients, Decision Trees (DTs) for predicting if a Covid-19 patient will eventually pass away by analyzing its clinical data, and a neural system that integrates the previous ones using Hierarchical Probabilistic Logic Programs (HPLPs). Predicting if a Covid-19 patient will end in a critical condition is useful for managing the limited number of intensive care at the hospital. Moreover, knowing early that a Covid-19 patient could end in serious conditions allows doctors to gain early knowledge on patients and provide special treatment to those predicted to finish in critical conditions. The proposed system, entitled Neural HPLP, obtains good performance in terms of area under the receiver operating characteristic and precision curves with values of about 0.96 for both metrics. Therefore, with Neural HPLP, it is possible not only to efficiently predict if Covid-19 patients will end in severe conditions but also possible to provide an explanation of the prediction. This makes Neural HPLP explainable, interpretable, and reliable.

**Graphical abstract:**
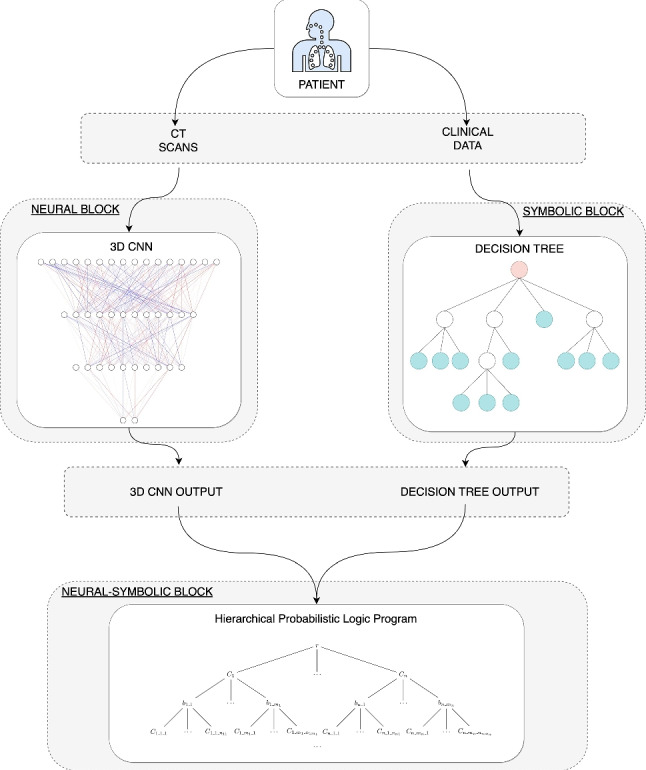
Representation of Neural HPLP. From top to bottom, the two different types of data collected from the same patient and used in this project are represented. This data feeds the two different machine learning systems and the integration of the two systems using Hierarchical Probabilistic Logic Program.

Representation of Neural HPLP. From top to bottom, the two different types of data collected from the same patient and used in this project are represented. This data feeds the two different machine learning systems and the integration of the two systems using Hierarchical Probabilistic Logic Program.

## Introduction

The global emergency caused by the spread of Covid-19 has highlighted the necessity for early-stage identification of complications and risk status of patients caused by the Covid-19 infection. This is because early diagnosis is vital for Covid-19 positive patients [1]. Thanks to the huge amount of data and much research on healthcare (Medicine 4.0), Artificial Intelligence (AI) technologies are increasingly applied to medical field [2–4]. Predicting complications of a certain disease by analyzing medical records of patients is hindered by many problems such as difficulty in finding patterns in structured clinical data, missing values, and a lack of annotation. For these reasons, predicting the risk of developing complications in the medical field is a relevant challenge. Currently, the analytical capability of Deep Learning (DL) algorithms has proven to be extremely accurate but not interpretable, understandable and therefore often not reliable. It is therefore necessary to build systems that are able to provide clear explanations of their decisions [5, 6] particularly in sensitive areas such as medicine. More importantly, it is necessary to motivate medical diagnoses or decisions with detailed reasoning and explanations. Due to the current historical period and thanks to the wide availability of data, applying ML and DL to Covid-19 data is an active and ongoing area of research [7, 8]. In this paper, neural and symbolic approaches to AI are investigated. Neural models, that belongs to DL family, are used to analyze unstructured data like Computed Tomography (CT) scans and symbolic models are used to analyze structured clinical data. The aim of this work is to design and implement a neural-symbolic model that is able to predict the severity of Covid-19 patients from clinical data and lung CT scans and enable the model to provide an explanation of its prediction. The idea is to extract relevant patterns from heterogeneous data collected from patients to produce a more comprehensive analysis.

The rest of the paper is organized as follows: Section 2 describes the adopted method and presents the different medical data used in the present work. Experiments on Decision Trees (DTs), 3D Convolutional Neural Networks (3D-CNNs), and Neural Hierarchical Probabilistic Logic Programs (Neural HPLPs) are presented in Section 3. Section 4 presents the obtained results. Section 5 discusses the proposed approach with some related work and finally, Section 6 concludes the paper.

## Methods

**Fig. 1:**
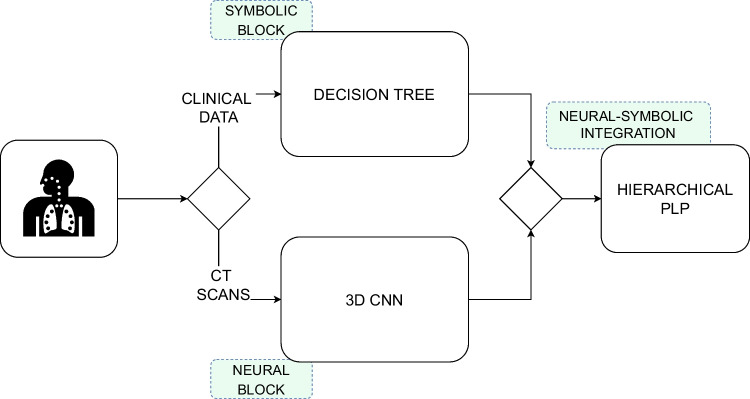
Neural-Symbolic Integration system: DT and 3D-CNN are integrated using HPLP

In order to predict the health state of Covid-19 patients arriving at the hospital, we propose a novel Neural-Symbolic method shown in Fig. 1, which integrates both symbolic, Probabilistic programs [9–11] and neural systems [12–14]. The neural-symbolic block is based on Hierarchical Probabilistic Logic Programming (Hierarchical PLP) [15], which is an ML model that is able to build scalable, reliable and explainable AI systems. HPLP receives as input the integration of the outputs of a DT system that predicts the severity state of Covid-19 patients from clinical data and a 3D-CNN that predicts the patients’ lungs state using lung CT scans. Then, HPLP learns a set of probabilistic rules that predicts, at an early stage, if a Covid-19 patient arriving at the hospital will end in a critical condition. Therefore, we trained a 3D-CNN for predicting the severity of lung lesions and a DT to predict the probability of a patient’s death during hospitalization. The output of these two systems is combined to generate the dataset for the final part of the system which integrates the neural and the symbolic approaches through HPLP.

### Dataset

The dataset is composed of two sub-datasets: clinical data and lung CT scans. The clinical dataset was provided by a hospital in Ferrara, Italy. It contains records of 502 Covid-19 positive patients collected during spring 2020 from which 126 died during hospitalization. Thus, the dead patients correspond to about 25*%* of the whole dataset. Each patient in the dataset has 59 clinical attributes. Additionally, 96 of the patients also had an associated CT scan. The 96 patients were kept as the test set. Of these 96 patients, 30 passed away during the hospitalization period. Table 3 in the Appendix shows the clinical attributes of each patient with the corresponding acronyms.

**Fig. 2:**
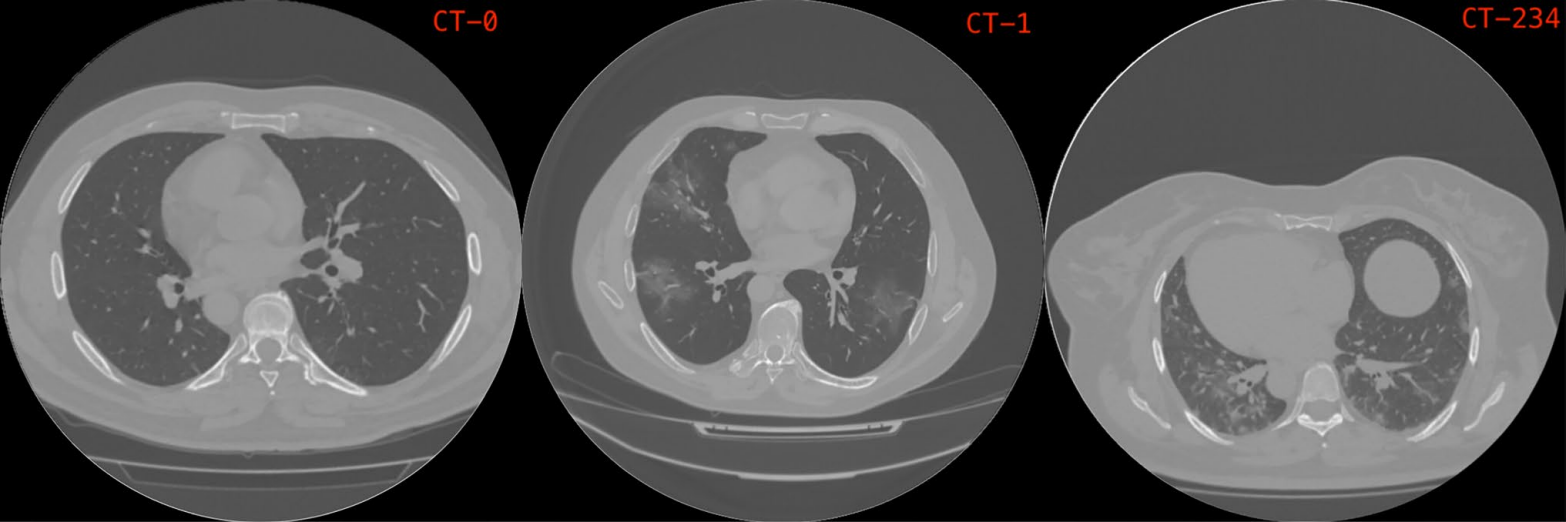
Example of images of a slide of a DICOM voxel for the three classes. From left to right images belonging to class CT-0, CT-1 and CT-234 respectively

The CT scans dataset is described in *MosMedData* [16]. It contains human lung CT scans with Covid-19-related findings, as well as without such findings. The CT scans were collected in 2020 and provided by the municipal hospital in Moscow, Russia. The dataset contains CT scans divided by the severity of lung tissue abnormalities with Covid-19. There are five classes: without injuries, with mild, moderate, severe and critical injuries respectively. The dataset is distributed as follows: CT-0, CT-1, CT-2, CT-3 and CT-4 contain 254, 684, 125, 45 and 2 patients respectively. It can be observed that the dataset is unbalanced towards the CT-1 class, and the mild injuries class. Due to the reduced numbers of the last three classes, they were merged into one class obtaining the following distribution: CT-0 with 254 (22.8%), CT-1 with 684 (61.6%) and CT-234 with 172 (15.6%) patients respectively. Figure 2 shows an example of an image for each class. These classes correspond to three different levels of severity of lung injuries that are as follows: *healthy*, *minor* and *serious*. We used as test set the CT scans of the 96 patients named previously. All images in this dataset are in *Digital Imaging and COmmunications in Medicine* (DICOM) format. So, a CT scan in DICOM format can be seen as a set of consecutive images that form a 3D image. For this reason, we used a convolutional neural network with 3D filters.

## Experiments

### Experiments on clinical data

In this experiment, done on the clinical dataset, see Section 2.1, a ML model that predicts the probability of a patient’s death during the hospitalization period is built. To balance the data, the *Synthetic Minority Oversampling Technique* (SMOTE) [17, 18] was applied. SMOTE selects a minority class instance and picks its *n* nearest neighbors belonging to the same class. The generated synthetic instance is then created by choosing one of the *n* nearest neighbors and connecting them with the chosen real instance to form a line segment in the feature space. Then, SMOTE is used to oversample the class of dead patients since it corresponds to 25% of the dataset. The ML models used for these experiments were DTs [19] and *Random Forests* (RFs) [20, 21]. The experiment is divided into two steps: the first step uses a RF to extract the most relevant clinical features that determine the patient’s death during hospitalization and the second step trains a DT using only the relevant features previously extracted. In fact, a new version of the dataset was created with the same number of patients but with only 10 clinical parameters. This new dataset was used to train a DT whose accuracy was similar to the one provided by the RF. A DT was used because it is possible to extract the entire decision path (in the form of a rule, see Rule 1) which provides an initial explanation of the prediction.

1 $$\mathbf{if}\;condition_1\;\wedge\; condition_2\;\wedge\;...\;\wedge\; condition_n\mathbf{then}\;outcome$$

The most relevant clinical attributes extracted by the RF, with an accuracy of $\sim 80\%$, are as follows: Age, Sex, Glomerular Filtration Rate (GFR), C-reactive Protein (CRP), Troponin, Creatinine, Lactate Dehydrogenase (LDH), Brain Natriuretic Peptide (BNP), Procalcitonin (PCT), White Blood Cells (WBC), Charlson Index. This result is in line with the work done by Yan li et al. [22] which states that LDH, lymphocytes and CRP are crucial predictive biomarkers of disease mortality with an accuracy of 90%.

After training a DT with the clinical attributes listed above, we achieved about 70*%* accuracy on the test set (i.e., on the 96 patients described at the beginning of this section).

### Experiment on lung CT scans

**Fig. 3:**
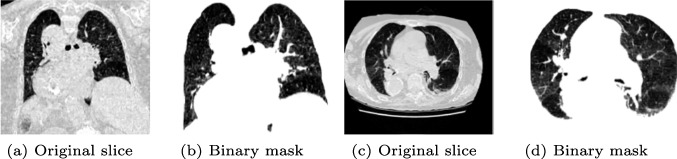
Segmentation of CT scans. The odd images represent an original slice of the DICOM voxel that depict the lungs of the patient. The even images represent the binary masks obtained after the pre-processing

The second experiment was performed on patient’s lung CT scans dataset, see Section 2.1. A deep neural network that predicts the gravity of lung injuries from patients’ CT scans is implemented. Before training the model, the CT scans were pre-processed using a segmentation that creates a lung’s binary mask followed by an application of a mask to eliminate unnecessary parts of the images, see Fig. 3. The segmentation was done using the *Hounsfield* (HU) scale. The HU scale is a quantitative scale for describing radiodensity in medical CT. On HU scale, air is represented by a value of − 1000 and bone between + 700 to + 3000. As bones are much denser than the other soft tissues, they show up much better in CT scans. Using this information, it was possible to identify which part of the image contains lungs and create a binary mask, lungs are represented by a value between − 700 to − 600 in the HU scale. After the segmentation and the application of the binary mask, the images were normalized between 0 and 1. It should be noted that the use of a fixed threshold for the segmentation of lungs, might be affected by different scanners and acquisition conditions [23]. This problem can be addressed via techniques based on unsupervised Fuzzy C-Means (FCM) clustering called spatial FCM (sFCM) [24]. Fundamentally, the FCM method [25] is a partitional clustering technique that minimizes the intra-cluster variance, as well as maximizes the inter-cluster variance, in terms of a distance metric between the feature vectors [26]. The FCM clustering does not take into account any spatial relationship among pixels since all the samples are used as dispersed and independent points. The sFCM [27] enables the retention of the same formulation and objective function as the classic FCM algorithm, just by modifying the update rules with the local spatial content in the image.

When working on 3D scans, in addition to the spatial characteristics of the images, the volumetric aspect of the CT scans has to be learned. The network trained in this work is a 3D-CNN composed as follows: two blocks with two 3D convolutional layers with 5 × 5 × 5 kernel and ReLU -like activation function followed by a max pooling layer with 98 and 160 neurons respectively. These two blocks are followed by two fully connected layers. The first with 110 neurons and the second is the output layer with 3 neurons corresponding to the three classes: CT-0, CT-1, CT-234.

The 3D-CNN was trained and validated on the MosMedData dataset achieving $\sim 70\%$ accuracy on the validation set. It was also tested on the CT scans of the 96 patients described in Section 3.1 achieving $\sim 54\%$ accuracy. This result is heavily conditioned by the low amount of CT scans in the dataset.

### Neural hierarchical probabilistic logic program (Neural HPLP)

In this section, a neural-symbolic system that allows easy integration of both symbolic and sub-symbolic models is proposed. It allows to build an efficient, interpretable and explainable system for early-stage prediction of the critical state of Covid-19 patients. The proposed system relies on HPLP [10, 15, 28–30], an extension of Liftable PLP [31], which is a recent AI approach for integrating symbolic (e.g., PLP) and sub-symbolic (e.g., neural networks) approaches of AI. The proposed system, named Neural HPLP, learns a predicate, also called target predicate using a set of examples called interpretations. Each interpretation is associated with each patient and is composed of the outputs of the DT and the 3D-CNN described in Sections 3.1 and 3.2 respectively. How to generate the interpretations is described in Section 3.3.1. The target predicate is, for a Covid-19 patient, that being in a critical state.

Now, suppose we want to compute the probability of atoms^1^ for a target predicate *r* using a PLP. In particular, we want to compute the probability of a ground atom $r(\vec {t})$^2^, where $\vec {t}$ is a vector of terms^3^. We consider a specific form of PLP that defines *r* in terms of *input predicates* (their definition is given as input and is certain) and *hidden predicates*, defined by clauses of the program. Discrimination is done between input predicates, which encapsulate the input data and the background knowledge, and the target predicate, which is the predicate we are interested in predicting, i.e., in our case Covid-19 patient in a critical state. We introduce the notion of hidden predicates which are disjoint from input and target predicates. Each clause in the program has a single head atom annotated with a probability. Furthermore, the program is hierarchically defined so that it can be divided into layers. Each layer defines a set of hidden predicates in terms of predicates of the layer immediately below or in terms of input predicates. A generic clause *C* is of the form

$$C=p(\vec{X}):\pi:-\phi(\vec{X},\vec{Y}), b_{1}(\vec{X},\vec{Y}),\ldots,b_{m}(\vec{X},\vec{Y})$$

where $\phi (\vec {X},\vec {Y})$ is a conjunction of literals^4^ for the input predicates. The vector $\vec {X}$ represents variables appearing in the head of *C* and $\vec {Y}$ represents the variables introduced by input predicates. $b_{i}(\vec {X},\vec {Y})$ for *i* = 1,…,*m* is a literal built on a hidden predicate. Variables in $\vec {Y}$ are existentially quantified with scope of the body. Only literals for input predicates can introduce new variables into the clause. Moreover, all literals for hidden predicates must use the whole set of variables of the predicate in the head $\vec {X}$ and of input predicates $\vec {Y}$. Moreover, we require that the predicate of each $b_{i}(\vec {X},\vec {Y})$ does not appear elsewhere in the body of *C* or in the body of any other clause, i.e., each hidden predicate literal is unique in the program. We call Hierarchical PLP the language that admits only programs of this form [15]. A generic hierarchical program is defined as follows:

$$\begin{array}{lll} C_{1}=r(\vec{X}):\pi_{1}&:-&\phi_{1}, b_{1\_1},\ldots,b_{1\_m_{1}}\\ &\ldots&\\ C_{n}=r(\vec{X}):\pi_{n}&:-&\phi_{n}, b_{n\_1},\ldots,b_{n\_m_{n}}\\ C_{1\_1\_1}=r_{1\_1}(\vec{X}):\pi_{1\_1\_1}&:-& \phi_{1\_1\_1},b_{1\_1\_1\_1},\ldots,b_{1\_1\_1\_m_{111}}\\ &\ldots&\\ C_{1\_1\_{n_{11}}}=r_{1\_1}(\vec{X}):\pi_{1\_1\_{n_{11}}}&:-& \phi_{1\_1\_{n_{11}}},b_{1\_1\_n_{11}\_1},\ldots,b_{1\_1\_n_{11}\_m_{11n_{11}}}\\ &\ldots&\\ C_{n\_1\_1}=r_{n\_1}(\vec{X}):\pi_{n\_1\_1}&:-& \phi_{n\_1\_1},b_{n\_1\_1\_1},\ldots,b_{n\_1\_1\_m_{n11}}\\ &\ldots&\\ C_{n\_1\_n_{n1}}=r_{n\_1}(\vec{X}):\pi_{n\_1\_n_{n1}}&:-&\phi_{n\_1\_n_{n1}}, b_{n\_1\_n_{n1}\_1},\ldots,b_{n\_1\_n_{n1}\_m_{n1n_{n1}}}\\ &\ldots& \end{array}$$

where *r* is the target predicate and $r_{1\_1 {\ldots } \_n}$ is the predicate of $b_{1\_1 {\ldots } \_n}$, e.g., $r_{1\_1}$ and $r_{n\_1}$ are the predicates of $b_{1\_1}$ and $b_{n\_1}$ respectively. The bodies of the lowest layer of clauses are composed only of input predicates and do not contain hidden predicates. Note that here the variables were omitted except for rule heads.

**Fig. 4:**
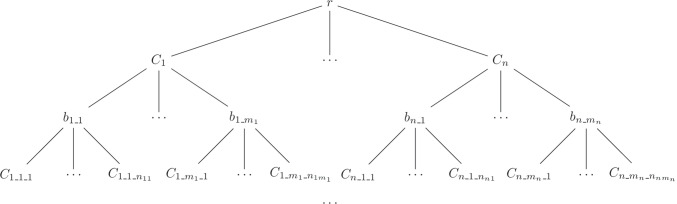
Generic Hierarchical Probabilistic Logic Program

A generic program can be represented by a tree, see Fig. 4 with a node for each clause and literal for hidden predicates. Each clause (literal) node is indicated with $C_{\vec {p}}$ ($b_{\vec {p}}$) where $\vec {p}$ is a sequence of integers encoding the path from the root to the node. The predicate of literal $b_{\vec {p}}$ is $r_{\vec {p}}$ which is different for every value of $\vec {p}$.

Given the target predicate to learn, i.e., a Covid-19 patient in a critical state, Neural HPLP learns from data a HPLP which consists of a set of logical clauses annotated with probabilities. The learned program is able not only to predict whether a patient arriving at the hospital will end in a critical state but it is also able to give a useful explanation of its prediction. To learn a HPLP, an algorithm entitled Structure LEArning of Hierarchical Probabilistic logic programming (SLEAHP) generates a set of clauses called *bottom clauses* from examples called interpretations. An *interpretation* is a whole description of a particular example. In our case it contains all clinical information concerning a patient, see Example 1. Then, an initially large HPLP is randomly generated from the bottom clauses. This large HPLP is converted into a deep neural network and algorithms such as Gradient Descent/Back-propagation, see [32], and Expectation Maximization, see [29], are applied to learn the probabilities associated with the clauses. Finally, clauses with very small values of probabilities are removed. For a detailed description of HPLP, see [15, 28]^5^. The following section described how to generate examples used to train the neural-symbolic block. Experiments predicting the critical state of a Covid-19 patient are described in Section 3.3.2

#### Data generation

Since Neural HPLP takes as input a set of interpretations that consists of the whole description of information regarding a single Covid-19 patient, we generated as many interpretations as the number of available patients by applying the following criteria: each interpretation is annotated with a predicate that defines the critical state of the corresponding patient: *a patient is in a critical state if the DT classifies him/her as subject to death soon (dead) or if the 3D-CNN classified its lung as in serious condition*. Two more predicates are added in the interpretation which correspond to the output of the DT (*dead* or *alive*) and the 3D-CNN (state of its lung, *serious*, *minor* or *healthy*) respectively. In order to enrich each interpretation, we also added in each the decision path, i.e., the set of predicates applied by the DT to take its decision, see Example 1.

##### *Example 1*

Consider the following interpretation that describes a Covid-19 patient with id 98:

$$\begin{array}{@{}rcl@{}} &&critic(98). \\ &&vital\_state(98,dead). \\ &&lung\_injury(98, minor). \\ &&age(98,94). \\ &&pcring(98,13.59). \\ &&ldhing(98,71.89). \\ &&troponina(98,0.0). \\ &&pcting(98,403.0). \\ \end{array}$$

where the first three predicates indicate that the patient was labelled as in critical conditional, the DT classifies him/her as dead and the 3D-CNN classifies his/her lung as in mild state. The other predicates are those included in the body of the decision path applied by the DT to predict the vital state of the patient, *vital*_*state*(98,*dead*).

#### Main experiments on neural HPLP

After training the DT and the 3D-CNN, the inference was performed on the corresponding test sets (96 patients) as described in Section 2.1. Classifications on the test set for both DT and 3D-CNN were compared with those given by an expert in the domain, a radiologist in particular. According to the expert, 51 were correctly classified. We then built 51 interpretations using the procedure described in the previous section. Among the interpretations, 20 were labelled as in a critical state and 31 as in a non-critical state. Given the reduced amount of data, the training procedure was done using cross-validation, i.e., the dataset is split into three folds with 17 interpretations in each fold. Every fold is balanced in terms of patients criticality. Interpretations in two folds are used for training and the remaining for testing. The procedure is repeated for the three crossed-combinations. Two versions of SLEAHP are applied: SLEAHP_DEEP which uses Gradient Descent/Back-propagation (specifically with Adam optimizer) for learning the parameters and SLEAHP_EM that uses Expectation Maximization as parameter learning. Both versions were trained with *L*_2 regularization [33–37] as described in [15], e.g., after learning, clauses annotated with probabilities less than a certain threshold are dropped. We used 10^− 5^ as threshold. Both algorithms were trained for 1000 iterations with early stop. The default Adam hyper-parameter was used in SLEAHP_DEEP.

### Additional experiments on neural HPLP

Before presenting the result of the present experiments in Section 4.1, an additional experiment was performed on a dataset similar to the one presented previously but this additional experiment was performed to assess Neural HPLP both on a limited and a consolidated dataset. The dataset used for the additional experiment was provided by Huazhong University of Science and Technology [38], Wuhan, China, and consists of 1521 patients of which 1126 from Union Hospital (HUST-UH) and 395 from Liyuan Hospital (HUST-LH). The dataset includes 894 Covid-19 positive patients (COVID^+^) and 627 non-Covid-19 patients (COVID^−^). All patients had 120 clinical attributes, and 1342 subjects had both CT and clinical data. To perform the experiments, patients with normal CT (class Normal) and with lung lesions (class Pneumonia) are considered. More precisely, 1006 patients with pneumonia and 336 patients with normal lungs. All examples in the dataset are in DICOM format. In the experiment, for each image, individual slices were extracted and processed. More precisely, only part of the images containing the lungs was considered. Table 4 in Appendix list all clinical attributes. A total of 47260 2D images were obtained and used for the training of a CNN. The dataset, grouped by the patient, was divided into training (75%), validation (10%), and testing (15%). Therefore, the test set includes 203 patients.

The trained CNN is composed of the following parts: four blocks composed of one convolutional layer with kernels of shape 3 × 3 and ReLU as activation function followed by a batch normalization layers with 64, 64, 128 and 256 neurons respectively. These blocks are followed by a global average pooling layer, one fully connected layer with 512 neurons and one dropout layer. The output layer consists of 2 neurons associated with the two classes: Normal, Pneumonia lung.

Regarding clinical data, a similar approach applied in the previous experiment, described in Section 3.1, is adopted. The only difference is that the RF and DT were used to predict COVID^+^ or COVID^−^ instead of the death of a patient during hospitalization.

## Results

This section presents the results on both the main and the additional experiments.

### Results on the main experiment

**Fig. 5:**
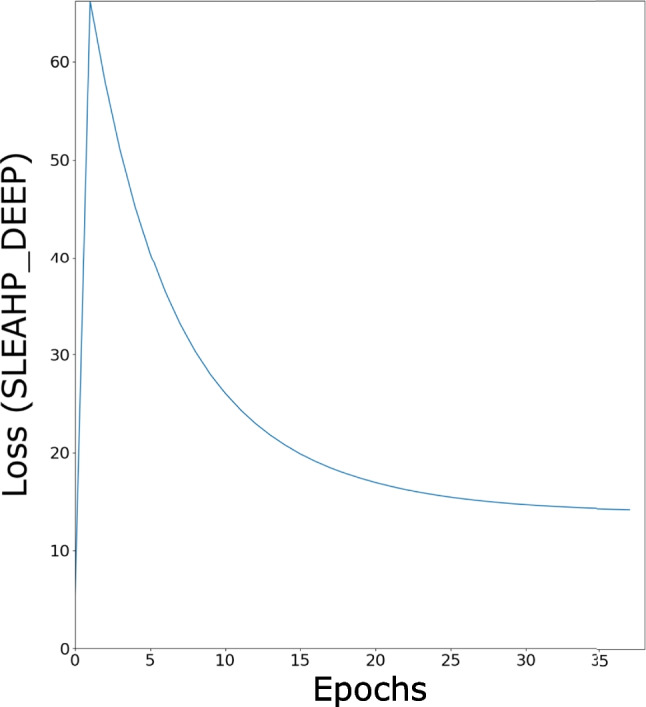
SLEAHP_DEEP loss function: training using the first two folds

**Fig. 6:**
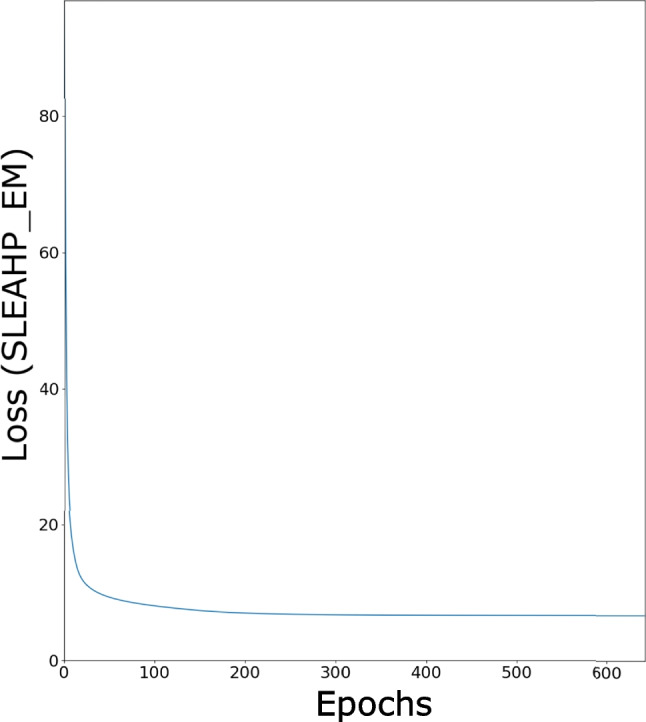
SLEAHP_EM loss function: training using the first two folds

This section presents the results of Neural HPLP. Since data used are unbalanced in both categories, we draw, for each test fold, the Receiver Operating Characteristics (ROC) and the Precision-Recall (PR) curves and compute the area under each curve (AUCROC and AUCPR) as described in [39]. The values of the areas, the final loss values and the associated average over the folds, highlighted in bold, for both SLEAHP_DEEP and SLEAHP_EM are shown in Tables 1 and 2 respectively. While these systems provide high performance both in terms of AUCROC and AUCPR, it is worth noting that SLEAHP_EM performs better than SLEAHP_DEEP. The perfect result obtained in Fold 3 was due to the fact that the combination of data included in folds 1 and 2 used for training was informative enough and enable the algorithm to learn a better theory. It could also be observed that the value of the loss function associated with Fold 3 is better than the ones associated with Fold 1 and 2. It also is worth noting that SLEAHP_EM converges faster than SLEAHP_DEEP as observed in Figs. 5 and 6.

**Table 1:** Areas under the curves and loss for SLEAHP_DEEP

	AUCROC	AUCPR	Loss
Fold 1	0.67347	0.80148	-10.80451
Fold 2	0.83333	0.90110	-8.99724
Fold 3	1.00000	1.00000	-5.57603
Average	**0.83560**	**0.90086**	**-8.45926**

**Table 2:** Areas under the curves and loss for SLEAHP_EM

	AUCROC	AUCPR	Loss
Fold 1	0.95918	0.95876	-4.64181
Fold 2	0.93750	0.94097	-5.45861
Fold 3	1.00000	1.00000	-4.17694
Average	**0.96556**	**0.9665766667**	**-4.75912**

An example of learned rules is shown in Example 2. From the example, it can be clearly highlighted the fact that the feature *pcting* is one of the most relevant clinical attributes useful to predict if a patient will end in a critical state. The first clause states that a Covid-19 patient is very likely to end in a critical state if his/her lungs are in a serious condition. This explanation is a clear consequence of the criteria for labelling interpretations defined in Section 3.3.1. Another interesting explanation can be observed using the combination of rules highlighted in bold: these rules state that if the troponina value of the Covid-19 patient is greater than 14.5, then the patient is very likely to end in a critical state. Similar explanations can be observed for the other clinical attributes. Based on the present work, doctors could pay more attention to these clinical values of a Covid-19 patient arriving at the hospital and improve their diagnosis and decision relying on the learned explanation.

#### *Example 2*

Learned rules for predicting the critical state of a Covid-19 patient critic:0.9983201826613162:-lung_injury(serious). critic:0.07404512050456119:-troponina(B). critic:0.0031878498774003394:-bnp(C),hidden_3(C). critic:0.009686597460037139:-age(D). critic:0.017233160198335595:-pcring(E). critic:0.9999999999999978:-pcting(F). critic:0.009842737641589272:-gender_f_2(G),hidden_8(G). critic:0.31365106628607303:-age(H),hidden_9(H). **critic: 0.9441254441618012:- troponina(I),hidden_10(I).** critic:0.0037801053824951802:-ldhing(J),hidden_12(J). critic:0.0037629815686686108:-charlsonindex(K),hidden_13(K). critic:0.00843829776843874:-age(L),hidden_1_4(L). critic:0.003175373172965623:-pcring(M),hidden_15(M). critic:0.025554356497234587:-ldhing(N),hidden_16(N). critic:0.009720608547637732:-gender_f_2(O),hidden_17(O). critic:0.20913363931689946:-age(P),hidden_18(P). critic:0.00023631655161687748:-pcring(Q),hidden_19(Q). critic:0.05738042137315996:-ldhing(R). critic:0.010041915381422406:-gender_f_2(S),hidden_21(S). hidden_3(C):0.00323811617948383:-greater_than(C,393.0). hidden_8(G):0.009921403222347414:-greater_than(G,2.0). hidden_9(H):0.31230871757212836:-greater_than(H,70.0). **hidden_10(I) :0.9441254441618012:- greater_than(I,14.5).** hidden_12(J):0.0037799297021227085:-greater_than(J,101.87). hidden_13(K):0.0037630849919179643:-greater_than(K,21.0). hidden_14(L):0.010897346883759151:-greater_than(L,78.0). hidden_15(M):0.0031347295724000745:-greater_than(M,22.79). hidden_16(N):2.911186251403075e-5:-greater_than(N,54.37). hidden_17(O):0.0084854038369232:-greater_than(O,2.0). hidden_18(P):0.20917254607034547:-greater_than(P,85.0). hidden_19(Q):0.043254912362177045:-greater_than(Q,7.82). hidden_21(S):0.009506938720927838:-greater_than(S,2.0).

### Results on the additional experiment

This section presents the results of Neural HPLP applied to a more consolidated dataset described in Section 3.4. This further experiment serves to confirm the reusability, validity and more importantly the efficiency of Neural HPLP. As mentioned in Section 3.4, the target is to identify patients positive to Covid-19. First, we trained a RF on all clinical data classifying if the patients are positive or negative to the Covid-19. As results, we obtained an accuracy of *93.9%, an AUCROC of 0.93 and an AUPRC of 0.86*. Then, using the trained RF, the first 10 (most important) clinical attributes are extracted and are the following: *Temperature*, *Coefficient variation of red cell volume distribution width*, *Standard deviation of red cell volume distribution width*, *Age*, *Lymphocyte count*, *Eosinophil percent*, *Eosinophil count*, *Neutrophil percent*, *Hemoglobin* and *Lymphocyte percent*. Considering only these features, a new dataset for training a DT is generated. After training the DT, the following metrics on the set are obtained: an *accuracy of 90.14%, an AUCROC of 0.9045 and an AUCPR of 0.9208*. Experiments on the trained CNN achieved the following results on the test set: an accuracy of 81.77%, an AUCROC of 0.823 and an AUCPR of 0.8709.

The last part of the experiment is performed using SLEAHP_EM and SLEAHP_DEEP. From the outcome of the DT and CNN, a dataset consisting of 203 interpretations (one for each patient in the test set) is generated for training SLEAHP systems. From the experiment, the following results are obtained: SLEAHP_DEEP achieved an AUCROC of 0.8188 and an AUCPR of 0.7210 while *SLEAHP_EM achieved better results with an AUCROC of 0.8956 and an AUCPR of 0.8144*.

In summary, this additional experiment on a consolidated dataset confirms the accuracy and more importantly the effectiveness of Neural HPLP.

## Discussion

Different studies demonstrate that early diagnosing of Covid-19 considerably decreases its mortality rate [1]. Our work introduces an explainable AI system, Neural HPLP, that predicts if a Covid-19 will end in a severe condition and therefore will need intensive care or more intensive treatment. Predicting if a Covid-19 patient will end in a critical condition is useful in managing the pandemic and saving human lives. In the peak of the crisis with numerous Covid-19 patients in severe conditions, managing the limited number of intensive care in any hospital becomes vital. Knowing early that a Covid-19 patient could end in serious conditions has many advantages: it allows doctors to gain early knowledge on patients and provide special treatment to those predicted to finish in severe conditions. Moreover, it allows doctors to predict the future number of patients in intensive care and therefore enable an optimal distribution of those places with respect to other critical diseases. Finally, by providing a rules-based explanation of its prediction, e.g., the clinical attributes relevant to detect the severity condition as in Example 2, Neural HPLP not only guides doctors to provide special treatments to those patients, but appears to be a more interpretable and reliable predictive model.

Based on the format of the medical data such as structured clinical data, CT, radiographs, and ECG, it is possible to find in the literature different approaches and applications of ML and DL algorithms that analyze and create predictive models on Covid-19 positive patients. Regarding clinical data, Chansik An et al. [40] used different ML models to diagnose Covid-19 patients based on socio-demographic information and medical status, for the nationwide cohort of South Korea. Dan Assaf et al. [41] used DL, RF and DTs to improve the management of the pandemic through the optimization of both medical resources allocation and triage procedures. An Italian study conducted by Augusto Di Castelnuovo et al. [42] used ML algorithms to analyze clinical data of about 3000 Covid-19 patients. The work aim at identifying the underlying characteristics affecting Covid-19 patients who died during hospitalization. Another study conducted by Yan Li et al. [22] uses eXtreme Gradient Boosting (XGBoost) and DTs to find some decision rules to detect patients with the highest risk of mortality.

Concerning work on CT scans and/or chest X-Ray, Ardakani et al. built a ML system that evaluates radiological features of CT images collected from patients with Covid-19 and non-Covid-19 disease. They used different ML algorithms to find the computer-aided diagnosis system with the best performance in distinguishing Covid-19 patients from non-Covid-19 pneumonia. Alsharman et al. [43] used a CNN to detect Covid-19 on CT scans in the early stage of disease course. Albahli [44] highlighted the high performance of DNNs in detecting Covid-19 patients. His model reached 89% of accuracy on synthetic data produced by GAN-based model. Parnian Afshar et al. [45] try an alternative framework based on Capsule Networks [46] called COVID-CAPS that is capable of handling small datasets. COVID-CAPS achieved an accuracy of 95.7%, sensitivity of 90%, specificity of 95.8%, and Area Under the Curve (AUC) of 0.97. In [47], the authors propose an interesting approach, similar to Neural HPLP, that works on both clinical and image data for predicting Covid-19 severity. The paper developed a ML model to predict Covid-19 severities and a model to predict progression to critical disorder. These models were trained on radiomics features and clinical variables. The work accurately predicts Covid-19 severity and progression to critical illness from radiomics features joined with clinical attributes. Differently from Neural HPLP, the proposed models do not provide a clear explanation of its prediction.

Other work addressing Covid-19 themes is being done. For example, based on the intensive care unit (ICU), the work of Cheng, Fu-Yuan et al. [48] exploits ML to create a risk prioritization tool that predicts the ICU transfer within 24 hours. Another interesting work done by Montomoli et al. [49] exploits Extreme Gradient Boosting (XGBoost) algorithm to predict the increase or decrease in patients’ Sequential Organ Failure Assessment (SOFA) score on day 5 after ICU admission.

The novelty of Neural HPLP mainly lies in the possibility of obtaining an explanation from the whole system thanks to the HPLP. In systems that exploit a different form of data, when using neural networks, it is almost difficult to provide an explainable interpretation of the results due to their black box nature. This differentiates Neural HPLP from the other works.

## Conclusions

In this paper, we propose Neural HPLP a neural-symbolic system for early-stage prediction of critical states of Covid-19 patients. Neural HPLP integrates two ML models to build an efficient, interpretable, and explainable system for predicting the risk of developing complications in patients affected by Covid-19 infection. The system is made up of a symbolic part (DT) that predicts a patient’s death during hospitalization, a neural part (CNN) for predicting the severity of the patient’s lung lesions, and a probabilistic logic model that relies on the previous models to predict if a Covid-19 patient will end in a critical state and therefore will potentially need intensive care. The application of Neural HPLP to a similar and consolidated dataset confirmed its efficiency. The obtained results confirmed not only the reliability of Neural HPLP but also its interpretability. By the synergy of three ML approaches, Neural HPLP provides an accurate, understandable and reliable predictive model.

As future directions of work, we plan to integrate a method of automatic segmentation of CT scans to avoid using a fixed threshold on the HU scale when extracting the lungs from the images. Moreover, we plan to build an end-to-end training process of Neural HPLP based on a customized optimization function. In this way, the training process will propagate the HPLP loss back to the other system components and will enable a more efficient training process. To better improve the accuracy and efficiency of Neural HPLP, we also plan to integrate multiple other machine learning algorithms such as support vector machines using Hierarchical Probabilistic Logic Programming. Finally, we plan to investigate the scalability of Neural HPLP by applying it to a very large amount of clinical data.

## Appendix. : Clinical data

Table 3 represent the list of the clinical data of the first dataset described in the paper. Tables 4 represent the list of clinical data of the second dataset for the additional experiment described in the paper.

**Table 3:** Clinical data for the main experiment

Clinical attribute	Acronym
Age	-
Gender	-
Organization Cost Centre	CdcoUO
Intensification of care	-
Pneumology department	-
Anesthesia and resuscitation department	-
Clinical onset with fever	-
Hospitalization day	-
Discharge day	-
In-hospital days	-
Symptoms cardiopulmonary onset	-
Gastrointestinal onset symptoms	-
Systolic Blood Pressure at the entrance	SBP
Diastolic Blood Pressure at the entrance	DBP
Heart rate	-
Breath frequency	-
Body temperature	-
Modified Early Warning Score	MEWS
Partial pressure of oxygen in a gaseous environment	pO2
PO2 / FiO2 ratio	PF
High Resolution TC	HRTC
High Resolution TC per ground glass	HRTCpergrpoundglass
White blood cells	WBC
Lymphocytes	-
C-reactive Protein	CRP
Procalcitonin	PCT
Creatinine	-
Glomerular Filtration Rate	GFR
Lactate Dehydrogenase	LDH
Brain Natriuretic Peptide	BNP
Fibrinogen	-
D-Dimero	-
Isoamylase	-
Alanine Aminotransferase	ALT
Creatine Phosphokinase	CPK
Ferritin	-
Troponin	-
Smoking habit	-
Hypertension	-
Ischemic heart disease	-
Heart failure	-
IRCIIIIVV	-
ICTUSoTIA	-
Chronic Peripheral Obliterative Arteriopathy	AOCP
Chronic Obstructive Pulmonary Disease	COPD
Mild liver disease	-
Moderate liver disease	-
Peptic ulcer	-
AIDS	-
Hemiplegia	-
Localized or hematological neoplasm	-
Metastasis	-
Dementia	-
Charlson index	-
Microcythemia	-
Inflammatory Bowel Disease	IBD
Diabetes	-
Diabetes without organ damage	-
Diabetes with organ damage	-

**Table 4:** Clinical data for the additional experiment

Clinical attribute	Clinical attribute
Age	Alkaline phosphatase
Sex	Alanine aminotransferase
Temperature	Aspartate aminotransferase
malattie pregresse	Urea nitrogen
covid	Calcium
CT	Chlorine
Morbidity	Total carbon dioxide
Mortality	Creatinine
Erythrocyte sedimentation rate	Latitude-glutamyltransferase
C-reactive protein	Globulin
Procalcitonin	Potassium
Mean corpuscular hemoglobin concentration	Magnesium
Mean corpuscular hemoglobin	Sodium
Mean corpuscular volume	Phosphorus
Hematocrit	Total bilirubin
Hemoglobin	Serum total protein
Red blood cell	Uric acid
Platelet distribution width	Total cholesterol
Plateletcrit	Creatine kinase
Mean platelet volume	High density lipoprotein cholesterol
Platelet count	Lactate dehydrogenase
Basophil count	Triglyceride
Eosinophil count	Anion gap
Monocyte count	Direct bilirubin
Lymphocyte count	Glucose
Neutrophil count	Low density lipoprotein cholesterol
Basophil percent	Osmotic pressure
Eosinophil percent	Prealbumin
Monocyte percent	Total bile acids
Lymphocyte percent	Pseudo-hydroxybutyrate dehydrogenase
Neutrophil percent	Cystatin C
White blood cell	Leucine aminopeptidase
Platelet larger cell ratio	5’nucleotidase
Standard deviation of red cell volume distribution width	Homocysteine
Coefficient variation of red cell volume distribution width	Serum amyloid protein A
D-Dimer	Small density low density lipoprotein
Thrombin time	CD3+ T cell
Fibrinogen	CD4+ T cell
Activated partial thromboplastin time	CD8+ T cell
International normalization ratio	B lymphocyte
Prothrombin time	Natural killer cell
Albumin/Globulin ratio	CD4/CD8 ratio
Albumin	Interleukin-2
Interleukin-4	White blood cell count
Interleukin-6	Squamous epithelial cell
Interleukin-10	Viscose rayon
TNF-pseudo	Unclassified crystal
IFN-latitude	Specific gravity
Fibrin/fibrinogen degradation products	Complement C1q
Antithrombin III	Hyaline cast
B-type brain natriuretic peptide precursor	Pathological cast
Indirect bilirubin	pH
Fungi (1-3)-tail-D-glucan	Complement C3
Urea	Immunoglobulin M
High-sensitivity C-reactive protein	Immunoglobulin A
Red blood cell count	Immunoglobulin G
Non-squamous epithelial cell	Yeast
Choline esterase	Complement C4
Sialic acid	Lipase
Pseudo-L-Fucosidase	Anti-streptolysin O
Lipoprotein A	Rheumatoid factor
Apolipoprotein A1	Bacterial count
Apolipoprotein B	Lactic acid
Leukocyte mass	

## Footnotes

1 An *atom* is a predicate, *p*, applied to some terms

2 An expression (atom, literal, term or formula) is *ground* if it does not contain any variable

3 A *term* is a variable, a constant, or a functor, *f*, applied to terms, $f(t_{1}, t_{2}, \dots , t_{n})$.

4 A *literal* is an atom or its negation

5 An online version of the system is available at https://cplint.eu/e/phil/phil_examples.swinb. The manual is also available at https://arnaudfadja.github.io/phil
